# Parental Understanding of Their Child’s Germline Genomic Testing: Intent of Disclosure to Their Child and Family

**DOI:** 10.3390/jpm13121656

**Published:** 2023-11-28

**Authors:** Belinda N. Mandrell, Alise K. Blake, Katianne M. Howard Sharp, Jami S. Gattuso, Rose B. McGee, Lynn Harrison, Annastasia Ouma, Mary Caples, Liza-Marie Johnson, Kim E. Nichols

**Affiliations:** 1 Division of Nursing Research, Department of Pediatric Medicine, St. Jude Children’s Research Hospital, Memphis, TN 38105, USA; jami.gattuso@stjude.org (J.S.G.); mary.caples@stjude.org (M.C.); 2 Division of Cancer Predisposition, Department of Oncology, St. Jude Children’s Research Hospital, Memphis, TN 38105, USA; alise.blake@stjude.org (A.K.B.); rose.mcgee@stjude.org (R.B.M.); lynn.harrison@stjude.org (L.H.); annastasia.ouma@stjude.org (A.O.); kim.nichols@stjude.org (K.E.N.); 3 Department of Psychology, St. Jude Children’s Research Hospital, Memphis, TN 38105, USA; katianne.sharp@stjude.org; 4 Department of Oncology, St. Jude Children’s Research Hospital, Memphis, TN 38105, USA; liza.johnson@stjude.org

**Keywords:** pediatric cancer, genomic sequencing, knowledge, disclosure of results

## Abstract

Genomic testing is becoming increasingly common in the care of pediatric patients with cancer. Parental understanding of germline results and their intent and timing of results disclosure to their child and family may have significant implications on the family unit. The purpose of this study was to examine parental understanding of germline genomic results and plans for disclosure to their child and other relatives. Semi-structured interviews were conducted with 64 parents of children with cancer, approximately eight weeks after parents had received their child’s results. Parents of children with negative results (n = 20), positive results (n = 15), or variants of uncertain significance (n = 29), were interviewed. Fifty-three parents (83%) correctly identified their child’s results as negative, uncertain, or positive. Most parents had disclosed results to family members; however, only 11 parents (17%) acknowledged discussing results with their child. Most parents delayed disclosure due to the young age of their child at the time of testing. In summary, most parents appropriately described their child’s germline genomic results, yet few discussed the results with their child due to age. Families should be followed with supportive counseling to assist parents in the timing and content of result disclosure to their children.

## 1. Introduction

For pediatric patients diagnosed with cancer, paired germline genomic sequencing is evolving into a component of the routine diagnostic workup [1] and is changing our care of patients, given the short- and long-term implications for hereditary cancer risk. If a pathogenic germline variant is found, the patient and family may be impacted by increased cancer screening, increased risk for secondary cancers, potential risk to other family members, and for many variants, unclear guidance for surveillance [2]. Patients may also be found to harbor variants of unknown significance (VUS), which may cause uncertainty in understanding potential risks to their child and other family members [3]. Once a child is tested, it is important that the provider or genetic counselor who coordinated the testing reviews when and how the parent plans to discuss the results with the child, especially for children who were found to have a pathogenic (P) or likely pathogenic (LP) variant in a cancer-predisposing gene [4]. Equally important is the disclosure of negative and VUS results as many parents overestimate their child’s heritable risk of cancer based on the presence of a positive family cancer history [5].

Several studies have explored mothers’ disclosure of their own pathogenic *BRCA1/2* results to their children, including minors [6,7]. Most mothers positive for alterations in these genes describe the communication of their results as beneficial to family members and children [7,8]. Although mothers desire honest communication, most report a lack of education and support with results disclosure [9,10,11]. Beyond breast cancer, a recent study explored the communication patterns among parents who tested positive for hereditary cancer or Huntington’s disease and their children aged 15–17 years [10]. Minor children expressed a desire to know more and felt that their parents did not fully disclose their results, while parents expressed the need for time to process these results. Parents felt that additional resources on how to communicate in a language that is understandable to their children were needed. While much has been written regarding parent disclosure of their own germline results, less is known regarding parent disclosure of the child’s germline results to the child and other family members.

The process of communicating germline genomic sequencing results to children and adolescents with cancer is dependent in part upon parent preference for their minor child and has become a topic of interest. To discuss results in a meaningful way with their child, parents must also understand the results and their implications. Decision making and family communication surrounding germline genetic testing for hereditary cancer predisposition among children, adolescents, and young adults is understudied [12]. The aim of this study was to examine parental understanding of their child’s germline genomic sequencing results and associated results disclosure or intent to disclose to their child and other family members.

## 2. Methods

### 2.1. Study Design and Participants

Participants were recruited into the institutional review board-approved Genomes for Kids (G4K) research study from August 2015 to March 2017, with the option to participate in questionnaires and semi-structured qualitative interviews. Eligible participants were scheduled for a study introduction visit approximately 1–2 months after cancer diagnosis and included all children with newly diagnosed or relapsed/refractory cancers who had sufficient fresh frozen tumor tissue for analysis. Prior to the study introduction and after verbal consent, parents were asked to complete a questionnaire assessing baseline genetic/genomic knowledge (pretest). After completion of the pretest, a research study nurse, trained by genetic counselors and using a standardized script, provided parents with written materials describing the study. The pretest responses were used to educate parents on genetic concepts they may have been unfamiliar with.

A follow-up visit was scheduled for those who were interested in participating. During the follow-up visit, the research nurse assessed the family’s understanding of the study, detailing potential germline results (P/LP, VUS, and negative), and obtained consent. Parents acknowledge that P/LP variants would warrant testing of family members and lifelong cancer predisposition screening of the proband. After the consent visit, parents were asked to repeat the genetic/genomic assessment (posttest). The pretest-to-posttest comparison found an 11% increase (77.8% to 88.9%; *p* < 0.0001) in genetic knowledge in 121 parents with both time points [13].

A subset of patients underwent germline sequencing only when tumor biopsy was considered unsafe or not clinically indicated. Enrolled patients were offered clinical whole genome (WGS), exome (WES), and RNA sequencing of their tumor and WGS and WES of their germline tissues with comprehensive analysis and reporting of 156 hereditary cancer predisposition genes [1]. In addition to study consent, parents and patients ≥ 18 years consented to receiving or deferring the germline results.

Once germline results became available, they were disclosed to the parent(s) or consenting patient by a genetic counselor. Results were defined as negative (absence of a pathogenic or likely pathogenic cancer predisposition gene variant), P/LP (presence of a pathogenic or likely pathogenic cancer predisposition gene variant), or VUS (clinical significance of one or more variants is uncertain and may later be reclassified as pathogenic or benign).

To examine parental understanding of results and their intent and timing of result disclosure to their child and family, a subset of parents was given the opportunity to participate in qualitative interviews. Eligibility for interviews included participation in G4K, consenting to the exploratory interviews, and being an English-speaking parent or patient. Of the 309 participants enrolled in G4K, 64 consenting parents representing 64 families were randomly selected for interviews at eight weeks (±four weeks) after disclosure of their child’s germline results.

### 2.2. Data Analysis

Interviews were transcribed verbatim with transcription quality assurance. Parental responses specific to questions regarding understanding surrounding return of results (classification as negative, positive, or VUS) and child/family disclosure of results were extracted from the semi-structured interview transcript. Five coders with training in qualitative research methods used the constant comparative method for inductive content analysis [14,15] using MAXQDA software version 2020 plus (MAXQDA, Berlin, Germany). For the qualitative aim, the number of participants was determined for each group (negative, P/LP, VUS) by reaching saturation. Codes were derived from parental responses to describing their child’s germline result and disclosure of the result to the child, other children, and other family members. The coders used consensus conversations until an agreement was made for all codes assigned to the responses during content analysis.

### 2.3. Statistical Analysis

Demographic data and code frequencies are described with descriptive statistics including means and percentages. The frequency of each code was counted. If a code occurred multiple times in a response, the code was tallied once. For each question, multiple codes that overlapped in meaning were consolidated for the final themes.

## 3. Results

### 3.1. Study Cohort

Sixty-four parents were interviewed approximately eight weeks after disclosure of their child’s genomic germline results. The interviews were predominantly completed by the child’s mother (50; 78%). Most children were newly diagnosed with cancer (53; 86%), mean age of 7.7 years (0.7–22), 54.7% were male, 76.6% were white, and 41% were diagnosed with acute lymphoblastic (ALL)/myeloid leukemia. Of the 64 parents, 20 (31.3%) children had negative germline results with a mean age of 10.2 years (range 2.3 to 22), 15 (23.4%) children had P/PL germline results with a mean age of 5.7 years (range 0.7 to 18), and 29 (45.3%) children had VUS with a mean age of 7.1 years (range 0.85 to 18.6). (Table 1).

Of the fifteen patients with positive results, seven were diagnosed with bilateral retinoblastoma and had a P/LP variant in *RB1*; three with ALL had a P/LP variant in *PALB2*, *VHL*, and *MUTYH*; one with recurrent desmoid tumor had a P/LP variant in *FANCM*; one with recurrent astrocytoma had a P/LP variant in *P53* gene; and three with solid tumors had the following P/LP variants: one with optic glioma had a P/LP variant in *NF1*; one with Ewings sarcoma had a P/LP variant in *BAP1*; and one with neuroblastoma had a P/LP variant in *SMARCA4*.

### 3.2. Tell Me Your Understanding of Your Child’s Germline Result

Most parents (57; 89%) correctly defined their child’s genomic test results, providing the correct definition for negative, P/LP, and VUS results. Two of twenty parents could not recall or define their child’s negative results, two of fifteen parents could not recall or define their child’s P/LP results, and three of twenty-nine parents could not recall or define their child’s VUS results. Twenty-one (72%) of the twenty-nine parents correctly identified that one or more of their child’s VUS results may be reclassified in the future as P/LP or negative.

### 3.3. Tell Me about Any Talks You Have Had with Your Child, Other Children, or Other Family Members Regarding the Germline Results

The parents were then asked if they had talked with their affected child, other children, or other family members regarding the genomic result. Among the 64 parents, 47 (73%) of the parents had not disclosed the results to their child due to their young age with a mean of 4.9 years (0.7–13.2). Eleven of the parents (17%) had disclosed/discussed the result (negative, L/LP, or variant) with their child/young adult with a mean age of 12.5 years (8–22). Among these, there were two parents whose children harbored a P/LP result. One was the mother of a 12-year-old child with neuroblastoma found to have a P/LP *SMARCA4* variant. The second was a father whose 18-year-old daughter with Ewings sarcoma was found to carry a P/LP *BAP1* variant. A total of 21 (33%) parents had disclosed the results to their child’s siblings (when applicable) and 48 (75%) had disclosed them to other family members. (Table 2).

### 3.4. Themes Arising from Interviews

All parents of children with retinoblastoma mentioned the pathological gene during the interview and were less surprised by the positive results. They described early discussions with the child’s primary or ocular oncologist who informed them of the high likelihood of an underlying *RB1* germline mutation at the time of diagnosis.

*“Because he has it in both eyes, the doctor had prepared us. It would be a huge surprise if it were not positive, so there was not too much emotion in finding out”.*

Among parents whose child had positive results, 9 of 15 (60%) parents conveyed the positive germline results to family members and desired to better understand the family history as many parents pursued their own germline testing.

*“My brother is expecting a baby, so they are aware there is a possibility if this mutation comes from our side of the family”.*

*“We will let our family know if one of us is a carrier, or it is just some kind of by chance mutation”.*

An 18-year-old describes actions taken after P/LP disclosure to the patient and family.

*“I remember the genetic counselor telling me that I had a BAB1 gene mutation and I’m at risk for melanoma and kidney cancer. So, I will be followed. I think I was prepared for something to be positive because my mother has cancer. My mother has explained this to my sisters, my family has been tested and are waiting for their results”.*

Another parent expressed the stressful conversation that evolved when eliciting the family history from her husband’s family.

*“His parents told me I knew their family and they didn’t have any problems; they did not cooperate with me. I told them I would not ask any more questions and if they wanted more grandchildren, I needed to know the family history”.*

Parents whose child was found to have a VUS expressed concern about knowing how to present the results to the child. Five parents verbalized that they would not share the findings, due to the uncertainty of the result.

*“If we tell her, I want to wait until high school, we want her to be as normal as possible at the end of this. I don’t want to burden her”.*

*“I have only discussed the results with her father, her grandparents would freak out, like bad”.*

*“We have discussed with our children and told them, there doesn’t appear to be a genetic cause for his cancer”.*

*“I told her that the results were undetermined, and she asked what that meant, I told her that they are going to keep an eye on her for the next few years. But other than that, they weren’t going to change treatment and that she was fine”.*

Others expressed an understanding that the results may be reclassified later, and they expected to be recontacted should the results change.

*“We understand that if they find something relevant, they will contact us later”.*

*“As research becomes more fruitful, I understand that they will re-exam the variance and contact us if something is significant”.*

## 4. Discussion

In this study, we explored parental understanding and conveying of germline genetic test results to their child, other children, and other family members. We observed that most parents correctly classified their child’s germline genomic results as negative, P/LP, or VUS, yet only 17% disclosed the results to their child. Due to the young age of the child, many parents endorsed deferring result disclosure until a more developmentally appropriate age. One-third of the parents disclosed the results to the patients’ siblings and 75% disclosed them to other family members.

Young age is the most cited reason parents choose not to disclose genetic results, including developmental readiness and the emotional difficulty associated with disclosure. Valdez et al. [16] analyzed qualitative interviews of 14 parents whose child(ren) harbor germline *TP53* mutations associated with Li Fraumeni Syndrome (LFS). At the time of the interview, 7 (50%) parents had disclosed the child’s LFS diagnosis to 10 (59%) of the children. The remaining parents had not disclosed results due to parental discord, the young age of the child, or the death of the child. All parents endorsed the need for open communication and understood the importance of the child being proactive with cancer screening. Parents with LFS stated they wanted to provide more information to their children than the information they were given as children. Further, they desired to disclose their child’s positive results at what they considered age-appropriate times while endorsing worry and dread surrounding these conversations. Additionally, parents expressed a need for age-appropriate resources to assist in germline test result disclosure, as well as support in planning when and how to disclose results.

Early disclosure of cancer genetic testing results within a family leads to care planning and reproductive decision making, while delay leads to family discord [16]. Most of the disclosure literature describes factors associated with parental disclosure and timing of their own P/LP variant to their family and children. Parents often delay disclosure of their own results to their child, with 30% of parents delaying discussion for several months to years [17,18]. Consistent with our findings, the most frequent reasons are age and maturity of the child. Additionally, parents want time to adjust to their personal results before sharing and to better understand the perceived utility of information to the child and the perceived vulnerability or resilience of the child [17,18,19]. While we found that 75% of parents conveyed the genetic results of their child to other family members, it is estimated that as many as 20–40% of at-risk family members are not aware that a cancer predisposition gene has been found in the family [19,20]. Last, Asian and Black women have been found to be less likely to convey their own genetic results to their children and family, as compared with Whites and non-White Hispanics [21]. Therefore, race and ethnicity should be considered during disclosure conversations.

The challenge in reporting germline test results is the lack of evidence to inform “best” practices [22]. For clinical decisions regarding the minor child, concepts of informed consent include parental permission and child assent. It is reported that most children at 12 years of age are competent in making clinical research decisions [23]. Assent for disclosure of individual results should be offered to adolescents with adult-like decision capacity [24]. Boston Children’s Hospital Gene Partnership offers disclosure guidance depending on the age of the patient and parental preferences [25]. Disclosure is often based on parental preference for patients < 13 years of age. The age of 13 years is thought to be a time when most participants are developmentally ready to receive genetic results. For patients < 18 years, both parents and patient preferences for disclosure must agree. For patients 18 years and older, the return of results should be discussed at the time of consent with respect to the patient’s preferences. Further study should ask parents the age they perceive disclosure to be appropriate.

When conveying genetic results, children may have difficulty understanding complex information that is biological, numerical, and emotional. Parents of children who have cancer endorse the involvement of a genetic counselor in assisting with consenting and disclosure communication [26,27]. Given their expertise in counseling families, the genetic counselor can assist the family in communicating the results, recommending lifestyle behaviors to decrease cancer risk, and outlining a screening protocol for early cancer detection. Through the delivery of honest open communication, the parent can enhance the child’s knowledge of their pathogenic variant and dispel misconceptions, providing support during a time of physical and emotional change [28]. Toward this end, many might benefit from assistance provided by genetic counselors and other healthcare providers or by shared communication with these clinicians. To maximize the personal utility of cancer predisposition testing, our study suggests parents need help integrating their results into their family unit and communicating the result to the child at the developmentally appropriate time. If the goal of screening is to identify at-risk children, we must support families in meaning making around their test results so that they, and later the child, can make informed healthcare decisions based upon the results of testing. Last, parents should be supported before and after germline sequencing as distress and a lower parental quality of life has been reported in parents when revealing their child has a cancer predisposition variant [29]. Parents should be assessed for worry during the testing process, with appropriate psychosocial support.

This study has several limitations. For example, we did not explore the disclosure process between the parent and child as most children at the time of the study were too young to understand the information. It is also important to note that this was a highly selected sample with possible ascertainment bias as families who received information on genomics may have been more interested in participating in the study. In determining the parental understanding of the P/LP and VUS results, we failed to ask the parent to identify the specific gene and we did not probe their understanding of WES or WGS. Additionally, more than 75% of those interviewed were white, while those identifying as black were less likely to participate in the G4K study [30]. Finally, we did not ask whether parents might lean on other family members, providers, or genetic counselors to explain the results to their children. Future research will follow these families to better understand the timing and process of disclosure as the child matures.

## 5. Conclusions

Most parents interviewed understood their child’s germline testing results as negative, P/LP, or VUS. Parents desire to communicate genomic sequencing results with their children; however, they prefer that this conversation be carried out at a more appropriate age for the child. Parents understand that VUS results may be reclassified over time and expect to be updated regarding the reclassification. Institutions offering germline sequencing should provide parents and patients with the resources to understand their germline genomic results and assist in developing a plan of disclosure for current and any future results. Further, to best capitalize on the potential of germline genomic testing, cancer predisposition transition programs should be developed to support adolescents and young adults with positive germline results as they transition to adult care, ensuring they are best equipped to become their own self-advocates.

## Figures and Tables

**Table 1:** Patient demographic and clinical characteristics (N = 64).

Characteristic	N (%)
Age Mean [Range]	7.7 [0.7–22]
Negative Germline	10.2 [2.3–22]
Positive Germline	5.7 [0.7–18]
Variant Germline	7.1 [0.85–18.6]
**Sex**	
Female	29 (45.3)
Male	35 (54.7)
**Race**	
White	49 (76.6)
Black	9 (14.1)
Asian	2 (3.1)
Multiple	4 (6.3)
**Diagnosis**	
Leukemia	26 (41)
Solid Tumor	22 (34)
Brain Tumor	16 (25)

**Table 2:** Germline sequencing results and family communication.

Negative Germline Sequencing (n = 20)	Results Number of Parents (%)
No Communication with Child	10 (50%)
Communication with Child	4 (20%)
Communication with Sibling	10 (50%)
Communication with Other Family	14 (70%)
**Positive Germline Sequencing (n = 15)**	**Results** **Number of Parents (%)**
No Communication with Child	14 (93%)
Communication with Child	1 (7%)
Communication with Sibling	6 (40%)
Communication with Other Family	10 (66%)
**Variant of Unknown Significance (n = 29)**	**Results** **Number of Parents (%)**
No Communication with Child	23 (79%)
Communication with Child	6 (21%)
Communication with Sibling	5 (17%)
Communication with Other Family	24 (83%)

## Data Availability

Data is available within the article.
